# Brain glucose metabolism changes acutely following exercise and tracks with the systemic lactate response

**DOI:** 10.1002/alz.091743

**Published:** 2025-01-09

**Authors:** Zachary D. Green, Casey S. John, Anneka Blankenship, Paul J Kueck, Riley E Kemna, Chelsea N Johnson, Jonathan D Mahnken, Lauren Yoksh, Jeffrey M Burns, Joseph S. Donald, Eric D Vidoni, Jill K Morris

**Affiliations:** ^1^ University of Kansas Alzheimer’s Disease Research Center, Fairway, KS USA; ^2^ University of Kansas Medical Center, Kansas City, KS USA

## Abstract

**Background:**

Aerobic exercise may positively affect brain health, although relationships with cognitive change are mixed. This likely is due to individual differences in the systemic physiological response to exercise. However, the acute effects of exercise on brain metabolism and biomarker responses are not well characterized in older adults or cognitively impaired individuals.

**Method:**

The Acute Exercise Response on Brain Imaging and Cognition (AEROBIC) study is a randomized, controlled trial (NCT04299308). We enrolled older adults (60+years) who were cognitively healthy (n = 30) or cognitively impaired (n = 30) based on a clinical exam (telephone CDR). Study visits included a cardiorespiratory fitness test, Fluorodeoxyglucose [18F] PET (FDG‐PET) scans with timecourse blood draws during both rest and exercise, and resting MRI. Participants were randomized to both intensity (moderate; 45‐55% heart rate reserve (HRR) or higher (65‐75% HRR)) and PET visit order. Lactate and various exercise‐related biomarkers were measured. Each session concluded with cognitive testing (NIH Toolbox). Change in whole‐grey matter cerebral glucose metabolism from rest to exercise was registered as the primary outcome. We used general linear modeling to test for a primary effect of Condition (exercise vs. rest), as well as the effects of intensity and cognitive diagnosis, on the change in FDG‐PET SUVR and lactate AUC.

**Result:**

FDG‐PET SUVR differed significantly between exercise (1.045 ± .082) and rest (.985 ± .077) across the entire cohort (Diff = ‐.060, t(58) = 13.8, p < .001). The effect of exercise (F(1,54) = 193.086, p < .001) was not dependent on diagnosis or intensity. Exercise increased Lactate AUC (F(1,56) = 161.988, p < .001), an effect stronger in the Higher intensity (MD = 97.0±50.8) than in the Moderate intensity (MD = 40.3±27.5; t = ‐5.252, p<0.001) group. Change between exercise conditions for lactate AUC and FDG‐PET SUVR correlated (R^2^ = 0.179, p<.001).

**Conclusion:**

Acute exercise decreases brain glucose metabolism in both cognitively healthy and impaired older adults. This effect tracks with the systemic lactate response. This supports the hypothesis that increased lactate may spare glucose and be a key brain fuel during exercise. Additional work to directly measure brain lactate metabolism following exercise and understand the role of central and peripheral lactate metabolism is warranted.

